# Dynamic cross-talk analysis among TNF-R, TLR-4 and IL-1R signalings in TNFα-induced inflammatory responses

**DOI:** 10.1186/1755-8794-3-19

**Published:** 2010-05-24

**Authors:** Shih-Kuang Yang, Yu-Chao Wang, Chun-Cheih Chao, Yung-Jen Chuang, Chung-Yu Lan, Bor-Sen Chen

**Affiliations:** 1 Laboratory of Control and Systems Biology, Department of Electrical Engineering, National Tsing Hua University, Hsinchu, 30013, Taiwan; 2 Institute of Bioinformatics and Structural Biology, National Tsing Hua University, Hsinchu, 30013, Taiwan; 3 Department of Life Science, National Tsing Hua University, Hsinchu, 30013, Taiwan; 4 Institute of Molecular and Cellular Biology, National Tsing Hua University, Hsinchu, 30013, Taiwan

## Abstract

**Background:**

Development in systems biology research has accelerated in recent years, and the reconstructions for molecular networks can provide a global view to enable in-depth investigation on numerous system properties in biology. However, we still lack a systematic approach to reconstruct the dynamic protein-protein association networks at different time stages from high-throughput data to further analyze the possible cross-talks among different signaling/regulatory pathways.

**Methods:**

In this study we integrated protein-protein interactions from different databases to construct the rough protein-protein association networks (PPANs) during TNFα-induced inflammation. Next, the gene expression profiles of TNFα-induced HUVEC and a stochastic dynamic model were used to rebuild the significant PPANs at different time stages, reflecting the development and progression of endothelium inflammatory responses. A new cross-talk ranking method was used to evaluate the potential core elements in the related signaling pathways of toll-like receptor 4 (TLR-4) as well as receptors for tumor necrosis factor (TNF-R) and interleukin-1 (IL-1R).

**Results:**

The highly ranked cross-talks which are functionally relevant to the TNFα pathway were identified. A bow-tie structure was extracted from these cross-talk pathways, suggesting the robustness of network structure, the coordination of signal transduction and feedback control for efficient inflammatory responses to different stimuli. Further, several characteristics of signal transduction and feedback control were analyzed.

**Conclusions:**

A systematic approach based on a stochastic dynamic model is proposed to generate insight into the underlying defense mechanisms of inflammation via the construction of corresponding signaling networks upon specific stimuli. In addition, this systematic approach can be applied to other signaling networks under different conditions in different species. The algorithm and method proposed in this study could expedite prospective systems biology research when better experimental techniques for protein expression detection and microarray data with multiple sampling points become available in the future.

## Background

One main interest of molecular biologists is to understand the underlying mechanisms in a cell, including the synthesis of DNA, RNA and protein and how these molecules are regulated. In the last decade, researchers have uncovered a multitude of biological facts, such as protein 3D structures and genome sequences and organizations. However, this information is not sufficient to interpret the entire biological process and to understand its robustness, which is one of the fundamental properties of living systems at different cell levels [1]. Thus understanding how genes, proteins, and small molecules interact to form the functional modules and robust systems has become one of the major challenges in systems biology in recent years. With the advance of experimental techniques, many researchers have utilized high-throughput data such as DNA microarray, yeast two-hybrid assay, co-immunoprecipitation, and ChIP-chip approach to study the bio-molecular networks. In particular, these kinds of data are usually integrated to construct various types of molecular networks including protein-protein interaction networks, gene regulatory networks, metabolic networks and gene co-expression networks. These molecular networks have been demonstrated with great potentials to discover basic functions and to reveal essential mechanisms for various biological phenomena, by understanding biological systems not on an individual component level but on a system-wide level [2].

One of the extensively investigated biological systems is the inflammatory system of humans. It orchestrates a complex biological process, which engages a variety of cell types that eliminate invading microorganisms to protect the host [3]. Infected hosts recognize the ligands on the surface of disease-causing pathogens and mobilize specific inflammatory defense mechanisms. On the other hand, pathogens can proactively perturb host defense signaling pathways to enhance their survival [4]. In this case, the hosts and the pathogens in the inflammatory responses are considered as two players with conflicts of interest in the game theory [5]. Therefore, the inflammatory responses are highly context dependent, suggesting unplumbed complexity, and a wealth of intricate intra- and inter-cellular interactions [6]. In the human self-protection mechanism, vascular endothelium plays a central role in the regulation of several inflammatory functions. Products from bacteria and viruses that stimulate leukocyte and endothelial release of cytokines, chemokines, and lipid mediators may also play a role in inflammation. These stimuli can alter gene regulation of endothelial leukocyte adhesion molecules, cell signal transduction pathways, and endothelial permeability. Tumor necrosis factor (TNF) is one of the important cytokine that has long been considered as a pathological factor implicated in the pathology of dozens of human diseases, including septic shock [7], cancer [8], rheumatoid arthritis [9], malaria [10] and other afflictions. Two protein families have been implicated in the signaling pathway mediated by the receptors for tumor necrosis factor (TNF-R). These include the death domain-containing proteins (TNFR1, TRADD, RIP and FADD) and the TRAF domain-containing proteins (TNFR2, CD40, and TRAF1-6). Based on current models, upon binding of TNF to TNFR1, a protein called the silencer of death domain (SODD) is released and TRADD is recruited. TRADD then recruits TRAF2, RIP, and FADD, leading to activation of signaling cascades that mediate c-jun N-terminal kinase (JNK) activation, nuclear factor kappa-B (NF-κB) activation, and apoptosis. Binding of TNF to TNFR2 expressed by endothelial cells may lead to cell activation or apoptosis. Because both responses are initiated by ligand binding to a single receptor, it is clear that TNF activates multiple signal transductions [11].

Apart from TNF-Rs, there are two other central signaling pathways mediated by toll-like receptor 4 (TLR-4) and interleukin-1 receptor (IL-1R) which both play important roles in inflammation by regulating the activity of transcription factors such as NF-κB. From TLR-4, IL-1R and TNF-R signalings to NF-κB, there is a convergence on a common IκB kinase complex that phosphorylates the NF-κB inhibitory protein IκBα, namely inhibitor of nuclear factor kappa-B kinase (IKK) [12,13]. Although how TNF-R signals on the cell surface activate the IKK complex is not completely understood, studies have identified most key signaling components, and uncovered post-translational modification and cellular translocation of these components [14]. Previously published studies have shown that upstream signaling components are mostly receptor-specific, but the principles of signaling are similar, involving the recruitment of specific adaptor proteins and the activation of kinase cascades in which protein-protein interactions are controlled by poly-ubiquitination [12]. Because they need to cope with a broader range of pathogens with limited resources, some efficient signaling structures are reduplicate. Therefore, understanding these pathways has been focused on the identification of signaling network, the role of cytokine inducer and the subcellular translocation of those components by integrating various types of genomic and proteomic data.

**Figure 1:**
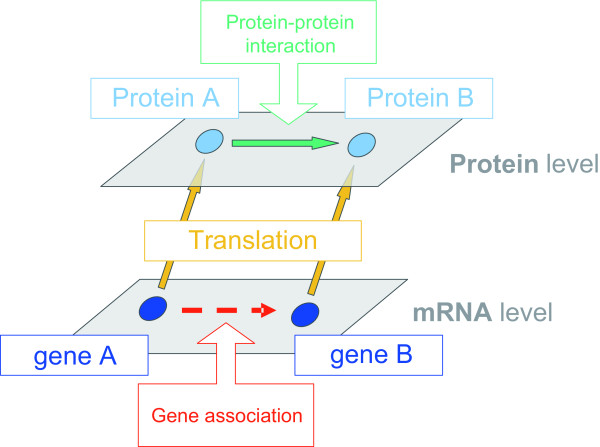
**Schematic diagram for reconstructing the protein-protein association**. This diagram shows the basic concept of the reconstruction of protein-protein association. On the protein level, the interactions between proteins from the well-known database and experimental data were extracted. However, this kind of interactions only reflects all possible static connections without stimulus-specific response or temporal changes. Our model includes the gene expression patterns from different time course to infer the dynamic protein-protein associations and networks, suggesting a more significant and realistic method for network reconstruction of the living organism.

Although many works have extracted some characteristics of TNF signaling transductions, however, there is still lack of a comprehensive approach to discover the systematic and dynamic properties of multi-pathway signaling networks under specific stimulant conditions. The most common way to construct protein-protein interaction (PPI) networks in the network studies is to use public available PPI database, published literatures and experimental data to connect the edges between proteins. However, most of these reconstructions of the PPI network merely displayed the static properties of interactions rather than discussing the dynamic associations and evolution of network topology. Therefore, the basic concept in this study lies in introducing the time-course gene expression data to endow the static protein-protein interactions with significantly dynamic changes, which correspond to the real circumstances in the living organism (see Figure 1). Various types of data were integrated, including gene expression profiles downloaded from the Gene Expression Omnibus (GEO) database (http://www.ncbi.nlm.nih.gov/geo/ accession number: GSE9055), pathway information from KEGG http://www.genome.ad.jp/kegg/[15] and NetPath http://www.netpath.org/[16], PPI information from BioGRID database http://www.thebiogrid.org/[17], and PPI clues from STRING http://string-db.org/[18] to infer a rough protein-protein association network (PPAN). According to the rough PPAN, a dynamic model was constructed for a protein to describe its associations with other proteins. Then, based on microarray expression profiles of different time stages, the association coefficients among these proteins were further identified by the constrained least squares parameter estimation method for each time stage, respectively. The insignificant associations were pruned and the significant protein-protein associations were reserved for the specific time stage according to the identified association coefficients. In this case, the preserved association represent the effective PPI for a specific time stage under a specific stimulus. This procedure was iterated one protein by one protein, and finally the whole protein-protein interaction networks were constructed for the vascular inflammatory response system at different time stages, which can be used to investigate the development of PPI network in the inflammation to the TNFα stimulus. A new cross-talk ranking method was also developed to evaluate the potential core elements in the related signaling pathways. Furthermore, a bow-tie structure was observed and considered as a core system mediating the input pathological factors and the output host responses for efficient processing of a broader range of pathogens with limited proteins and pathways, suggesting the robustness of the network.

## Methods

### Constructing the rough PPANs

**Figure 2:**
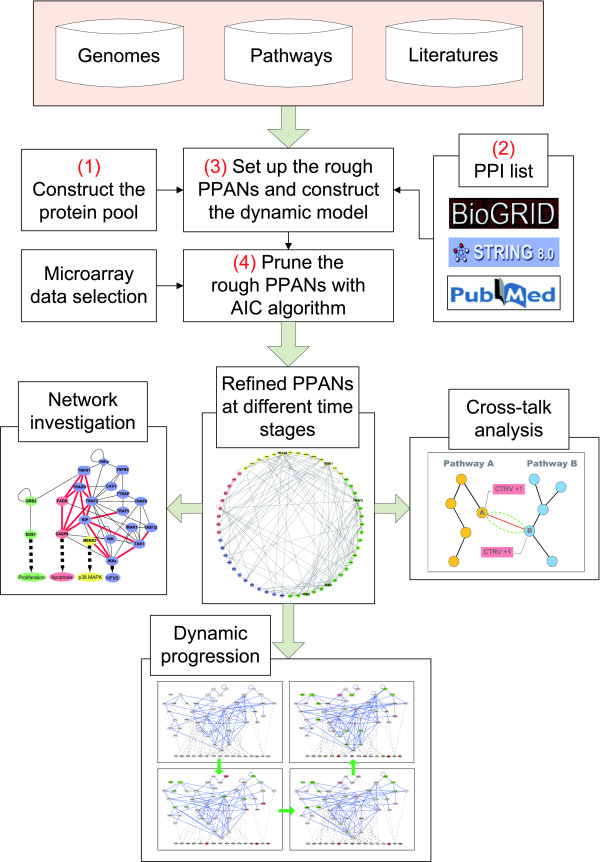
**Flowchart of the proposed method to construct the protein-protein association network (PPAN)**. This flowchart depicts the process to construct the protein-protein association network (PPAN) and the afterward investigations in this study. The four key steps of PPAN construction are described in details in the text. The rough PPAN is set up from steps (1) and (2), and the refinement is then performed in steps (3) and (4) to obtain the refined PPAN.

The dynamic expression and assembly of all functional components in the genome of an organism are significantly influenced by the environment. In this study, we aimed at the reconstruction of the protein-protein association networks under TNFα stimulus at different time stages and the analysis of dynamic cross-talks to investigate the network characteristics of an endothelial inflammatory system. The proposed method of the PPAN construction is divided into four steps (see Figure 2). The rough PPAN responsive to TNFα stress is established in the first two steps, and the refinement is then performed in the last two steps.

#### Step 1

In this step, the proteins of interest were selected to construct the protein pool. We first selected 21 proteins (see Table 1, column 1) that are associated with the TNFα stress based on data mining in the published literatures and known pathway diagrams [12,14-16,19-22]. Then, we selected 24 proteins which are involved in IL-1 and TLR-4 (both MyD88-dependant and MyD88-independant) pathways and might be triggered under the TNFα stimulus (see Table 1, columns 2-4). Finally, we chose 15 negative regulator proteins [14,23,24] which are considered to have anti-inflammatory therapeutic potential and/or possible feedback control signals in human endothelial inflammatory system (see Table 1, column 5). The total 60 proteins constituted the protein pool in this study and the protein-protein association networks were constructed among these proteins. Further, the Gene Ontology annotations for these 60 proteins are shown in Supplementary Table S1 [Additional file 1].

**Table 1:** Human protein candidates and their pathway catalogues in this study.

TNF	IL-1	MyD88-dependent TLR-4	MyD88-independent TLR-4	Negative regulators
TNF	IL1α	MyD88	TLR4	A20
TNFR1	IL1β	TIRAP	TRAM	CYLD
TNFR2	IL1R1	CD14	TRIF	FLN29
TRADD	IL1R2	PELI2	TRAF3	IRAK3
FADD	TOLLIP	IRAK4	TANK	NOD2
GRB2	ST2L	IRAK1	TBK1	RIP3
SOS1	PELI1	NIK	IKKε	PTPN11
CAV1	ECSIT	BCL10	IRF3	RNF216
CASP8				SARM
TRAF2				SIGIRR
TRAF5				SOCS1
TTRAP				TMED1
TRAF6				TNIP3
RIP				TRAF4
MEKK3				UBE2N
TAK1				
TAB1				
TAB2				
IKKα				
IKKβ				
IKKγ				

Proteins in the cross-talk analysis are classified into three major pathways, including TNFα, IL-1, TLR-4 (for both MyD88-dependent and MyD88-independent) pathways. The full names of these proteins are listed in the following. BCL10: B-cell CLL/lymphoma 10, NOD2: nucleotide-binding oligomerization domain containing 2, CASP8: caspase 8, apoptosis-related cysteine peptidase, CAV1: caveolin 1, caveolae protein, 22 kDa, CD14: CD14 molecule, IKKα: conserved helix-loop-helix ubiquitous kinase, CYLD: cylindromatosis (turban tumor syndrome), FADD: Fas (TNFRSF6)-associated via death domain, GRB2: growth factor receptor-bound protein 2, IKKβ: inhibitor of kappa light polypeptide gene enhancer in B-cells, kinase beta, IKKε: inhibitor of kappa light polypeptide gene enhancer in B-cells, kinase epsilon, IKKγ: inhibitor of kappa light polypeptide gene enhancer in B-cells, kinase gamma, IL1α: interleukin 1, alpha, IL1β: interleukin 1, beta, IL1R1: interleukin 1 receptor, type I, IL1R2: interleukin 1 receptor, type II, ST2L: interleukin 1 receptor-like 1, IRAK1: interleukin-1 receptor-associated kinase 1, IRAK3: Interleukin-1 receptor-associated kinase 3, IRAK4: interleukin-1 receptor-associated kinase 4, IRF3: interferon regulatory factor 3, NIK: mitogen-activated protein kinase kinase kinase 14, MEKK3: mitogen-activated protein kinase kinase kinase 3, TAK1: Mitogen-activated protein kinase kinase kinase 7, TAB1: mitogen-activated protein kinase kinase kinase 7 interacting protein 1, TAB2: mitogen-activated protein kinase kinase kinase 7 interacting protein 2, MyD88: myeloid differentiation primary response gene (88), PELI1: pellino homolog 1 (Drosophila), PELI2: pellino homolog 2 (Drosophila), PTPN11: protein tyrosine phosphatase, non-receptor type 11 (Noonan syndrome 1), RIP: receptor (TNFRSF)-interacting serine-threonine kinase 1, RIP3: receptor-interacting serine-threonine kinase 3, SARM: sterile alpha and TIR motif containing 1, SIGIRR: single immunoglobulin and toll-interleukin 1 receptor (TIR) domain, ECSIT: ECSIT homolog (Drosophila), SOCS1: suppressor of cytokine signaling 1, SOS1: Son of sevenless homolog 1 (Drosophila), TANK: TRAF family member-associated NFKB activator, TBK1: TANK-binding kinase 1, TIRAP: toll-interleukin 1 receptor (TIR) domain containing adaptor protein, TLR4: toll-like receptor 4, TMED1: transmembrane emp24 protein transport domain containing 1, TNF: tumor necrosis factor (TNF superfamily, member 2), A20: tumor necrosis factor, alpha-induced protein 3, TNFR1: tumor necrosis factor receptor superfamily, member 1A, TNFR2: tumor necrosis factor receptor superfamily, member 1B, TNIP3: TNFAIP3 interacting protein 3, TOLLIP: toll interacting protein, TRADD: TNFRSF1A-associated via death domain, TRAF2: TNF receptor-associated factor 2, TRAF3: TNF receptor-associated factor 3, TRAF4: TNF receptor-associated factor 4, TRAF5: TNF receptor-associated factor 5, TRAF6: TNF receptor-associated factor 6, FLN29: TRAF-type zinc finger domain containing 1, TRAM: toll-like receptor adaptor molecule 2, RNF216: TRIAD3 protein, TRIF: toll-like receptor adaptor molecule 1, TTRAP: TRAF and TNF receptor associated protein, UBE2N: ubiquitin-conjugating enzyme E2N (UBC13 homolog, yeast)

#### Step 2

To construct the static protein-protein interaction list for these 60 proteins, PPI information from BioGRID http://www.thebiogrid.org/[17] and STRING http://string-db.org/[18] databases were used. BioGRID (Biological General Repository for Interaction Datasets) contains over 198,000 of protein and genetic interactions information from six major model organism species [17]. STRING (the Search Tool for the Retrieval of Interacting Genes/Proteins) is a database of known and predicted protein interactions, including direct (physical) and indirect (functional) associations; they are derived from four various genome and high-throughput sources. STRING currently covers 2,483,276 proteins from 630 organisms [18]. These two databases were integrated to indicate the possible association between two proteins, and the rough PPAN was constructed based on the 60 proteins in the protein pool and the possible interactions among them.

### Pruning the rough PPAN via a dynamic model

On the basis of the database information and the literature evidences, the rough PPAN, which consists of all possible static interactions among the proteins of interest, was constructed. Since the rough PPAN only outlined the possible protein interactions under all kinds of experimental conditions, these interactions were further pruned to indicate the effective protein interactions under TNFα stress. As the time profiles of microarrays may reflect the co-existence of two particular proteins, time-course microarray data were employed to assess the significance of protein-protein interactions at certain time. Here, a dynamic regulatory model and model selection method Akaike Information Criterion (AIC) [25] were used to prune the rough PPAN. The details of the pruning process are described in the following paragraph and in Supplementary Methods [Additional file 2].

#### Step 3

In this step, the protein association equation was used to describe the protein-protein associations between target proteins of interest and its possible interacting proteins of the human inflammatory system. For a target protein *p *in the rough PPAN, the stochastic dynamic model of the protein is as follows [26](1)

where *y_p_*[*t*] represents the protein expression level at time *t *of the target protein *p*, *b_pq _*denotes the association parameter of the *q*-th interactive protein to *p*-th target protein, *y_q_*[*t*] represents the protein expression level of the *q*-th regulator protein interacting with the target protein *p*, *α_p _*denotes the effect of translation from mRNA to protein, *x_p_*[*t*] represents the mRNA expression level of the corresponding target protein *p*, *β_p _*indicates the degradation effect of the protein and *ω_p_*[*t*] is the stochastic noise. The biological meaning of equation 1 is that the protein expression of the target protein *p *at the next time *t*+1 is contributed by the concentration of protein *p *at the current time *t*, the effect of *Q *regulatory protein interactions, the translation effect from mRNA, the degradation effect of the present time, and some stochastic noises [26].

#### Step 4

Association parameters in equation 1 were identified in this step, and the model selection method Akaike Information Criterion (AIC) was further used to prune the rough PPAN according to the significance of the association parameters. By solving the constrained least squares parameter estimation problem and applying the most parsimonious model order detection using the AIC algorithm (see Supplementary Methods [Additional file 2]), we sieved out the regulatory proteins that significantly interact with the target protein on the genomic level, i.e. among *Q *proteins in the rough PPAN, only *Q' *proteins significantly interact with protein *p*. In other words, the insignificant protein interactions or non-involved protein association in this analytic time period could be deleted by AIC. The pruning process was repeated one protein by one protein. Consequently, the rough PPAN was pruned to become the refined PPAN.

### Cross-talk analysis by counting the CTRVs

**Figure 3:**
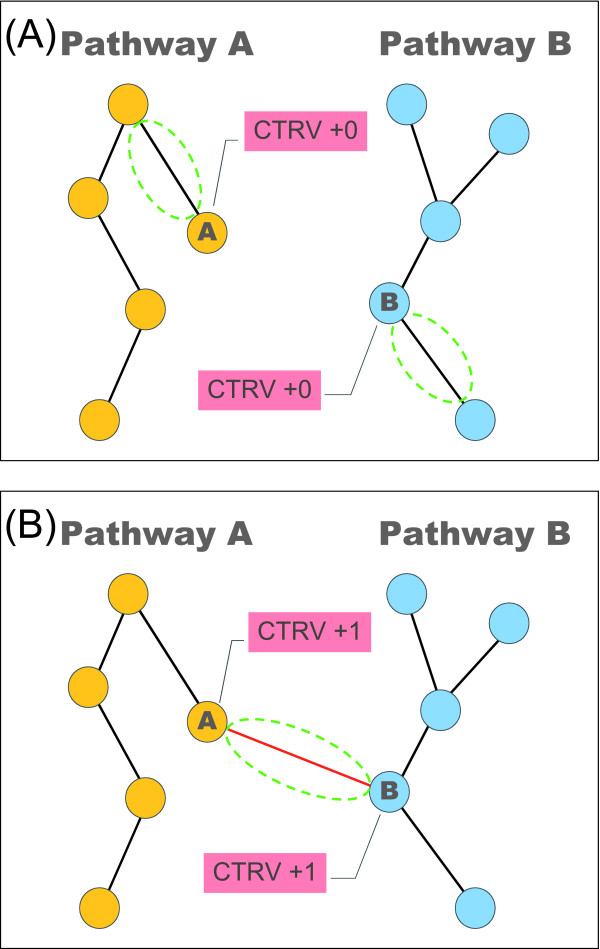
**Counting for the cross-talk ranking values (CTRVs)**. CTRV is a ranking value which reflects the potential of a protein in connection with multiple pathways. (A) All proteins connected with protein A belong to the same pathway A. In this case, the CTRV of protein A will not be changed. (B) If protein A connects with protein B, which belongs to a different pathway, then both CTRVs of protein A and B will be added by one.

To extract the significance from ever-changing associations and to determine if a protein is a possible cross-talk candidate, an original approach based on catalogues of pathway and dynamic associations in sequential time stages was proposed to compute the **C**ross-**T**alk **R**anking **V**alue (CTRV) for cross-talk analysis. We first clustered every protein (node) in the network into different pathways, and then calculated the number of associations which link outward to different pathways for each node. The numbers of associations in different time stages were summed up, resulting in the CTRVs. In general, if a protein has more associations to connect with several pathways, it would be considered to have more possibility as a cross-talk candidate. It should be noted that the protein associations, which link out as regulations to the other proteins and link in as regulations from other protein, are both important to the cross-talk analysis. A highly regulated protein always plays a critical role to receive, mediate or amplify signaling cascade, and a protein which intensively regulates others is usually characterized as an activator, inducer and transcription factor. Therefore, the proposed measurement of CTRV concentrates on counting the associations which link to different pathways for each protein, rather than takes account of every linkage as the ranking value, and the CTRVs of proteins are considered as a measurement to reflect the potential of a protein in connection with multiple signaling pathways. For example, protein A belongs to pathway A, and protein B belongs to pathway B. If all the proteins connected with protein A are within the pathway A, the numbers of connection for protein A will be considered as no change (see Figure 3A). If protein A connects with protein B but they belong to different pathways, then both ranking numbers of protein A and B will be added by one (see Figure 3B).

Because the CTRVs are counted based on the pathway catalogues, the way of pathway classification will be a critical step to determine the values of CTRVs. There are some proteins which have been proven to be involved in more than one pathway, but it is not appropriate to assign one protein to multiple pathway catalogues. The multi-assignment will decrease the values of CTRVs, which may cause the true cross-talk candidates to be neglected. To overcome this problem, every protein is only assigned to one pathway in this analysis. We assume that the active sequence under the TNFα stress is TNFα, IL-1, MyD88-dependent and finally MyD88-independent pathway. If there is a protein involved in more than one pathway, this protein will be assigned to the earliest activated pathway among them. Because the realistic situation for the activated sequence of pathways *in vivo *is unavailable, this hypothesis is based on the feedback signal from the known knowledge. In other words, TNFα stress will induce the gene expression and secretion of IL-1 cytokine [27], and upon binding of IL-1, the IL-1 receptor will associate with IL-1RAcP [28,29], forming a functional signaling receptor complex to involve MyD88 to activate the related pathway [30] Nevertheless, there is a lack of evidence to reveal that the TNFα stimulus will induce the MyD88-independent pathway, so we assume that this pathway is activated last.

## Results

### Construction of refined PPANs at different time stages of inflammatory system

**Figure 4:**
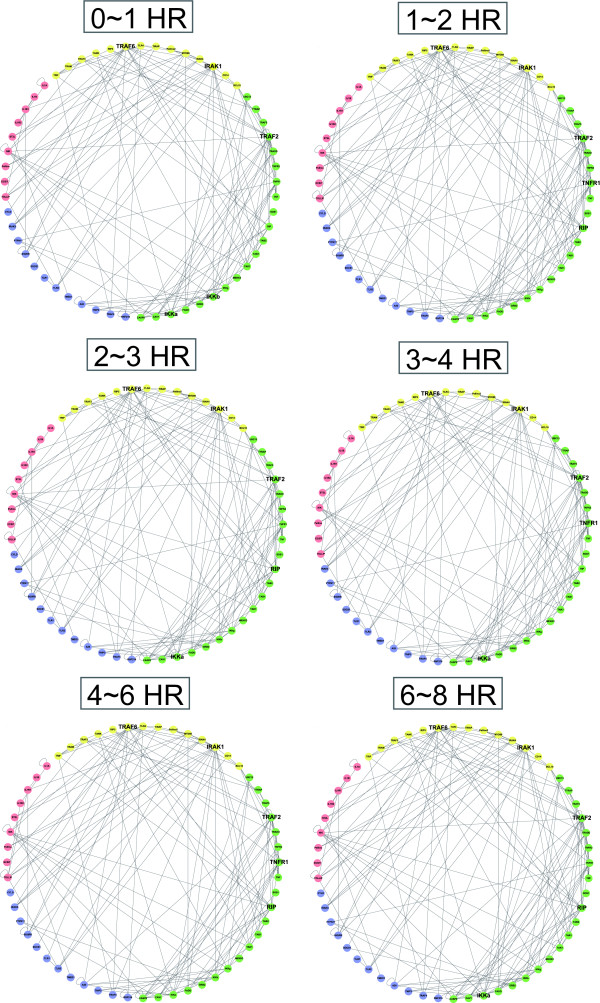
**Refined PPANs for HUVEC under TNFα stress at different time stages**. The illustration shows a time-series layout for each refined PPANs from 0 to 8 hour. Every refined PPAN is identified via a set of gene expression profile with five data points. In order to distinguish proteins involved in different signaling cascades, proteins belong to the same pathway are labeled with the same color. The progression of ever-changing associations obviously reveals that new connections continuously emerge, reflecting the fact that new signaling modules and function communities are involved in the endothelial inflammatory response to the TNFα stimulus. In addition, the top five hubs with highly connected degree are marked with larger font size. The numbers of nodes, edges and the highly connected hubs at different time stages are outlined in Table 2.

The proposed method was used to investigate the refined PPANs at different time stages of inflammatory system under TNFα stress. The genome-wide microarray data downloaded from the Gene Expression Omnibus (GEO) database at the NCBI website (http://www.ncbi.nlm.nih.gov/geo/ accession number: GSE9055) were adopted in this study. HUVEC (human umbilical vein endothelial cells) were treated with 10 ng/ml TNFα and the samples were collected every 15 or 30 minutes (0~8 hrs, 25 time points) [31]. The data set of 25 time points is divided into 6 time stages (0~1, 1~2, 2~3, 3~4, 4~6, 6~8 hr), resulting in 6 subsets of data. For each time stage, the corresponding data is used to identify the parameters in equation 1 and to prune down the rough PPAN based on the identified parameters to obtain the refined PPAN of each time stage, respectively. Consequently, six refined PPANs are constructed for six time stages. These refined PPANs are rearranged and visualized by the Cytoscape tool [32] (see Figure 4). The numbers of nodes, edges and the highly connected hubs at different time stages are shown in Table 2.

**Table 2:** Statistics of the TNFα-induced protein-protein association networks of HUVEC.

Duration	Nodes	Edges	Highly connected proteins				
**0~1HR**	56	144	TRAF2	IRAK1	TRAF6	IKKα	IKKβ
**1~2HR**	56	156	TRAF2	TRAF6	IRAK1	RIP	TNFR1
**2~3HR**	56	150	IRAK1	TRAF6	TRAF2	IKKα	RIP
**3~4HR**	55	152	TRAF2	IKKα	TNFR1	IRAK1	TRAF6
**4~6HR**	56	151	IRAK1	TRAF2	TRAF6	RIP	TNFR1
**6~8HR**	56	156	TRAF2	TRAF6	RIP	IKKα	IRAK1

Summary of the reconstruction of protein-protein association networks at different time stages. Proteins with maximal protein-protein associations are sorted in this table. These proteins are usually considered as the hubs which will be involved in several biological functions and play important roles in the signaling network.

### Investigation of the TNFα signaling network

**Figure 5:**
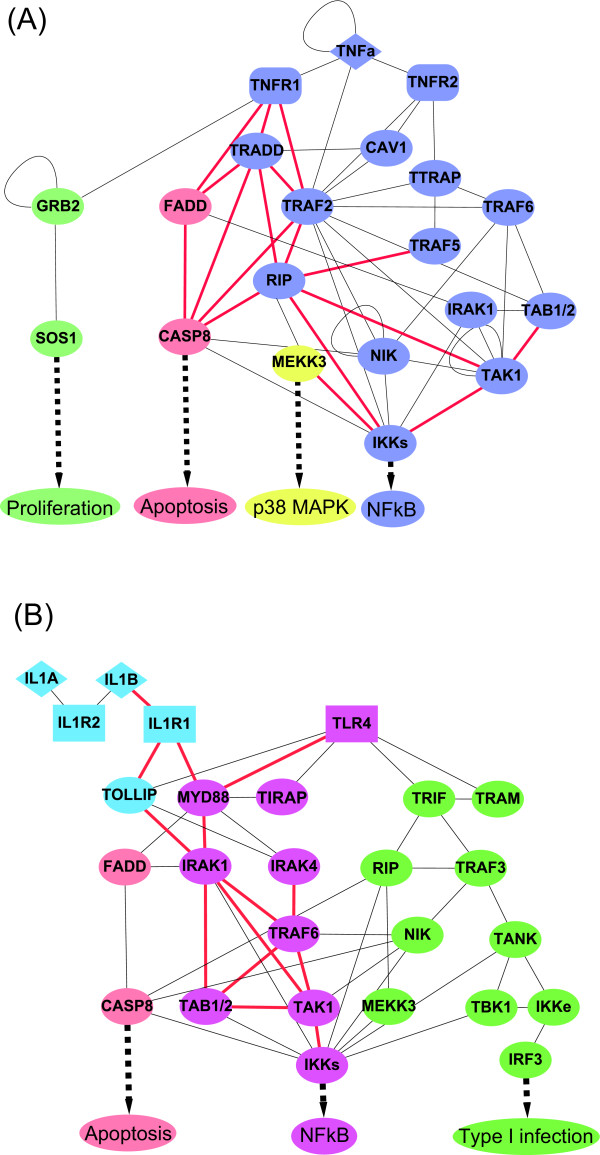
**Investigations of the (A) TNFα-related pathway and (B) IL-1/TLR4-related pathways**. Protein components which are ultimately responsible for the trigger of an inflammatory function are labeled with the same color in the pathway, and the thicker red edges represent the associations listed in Supplementary Table S2 [Additional file 3] and Supplementary Table S3 [Additional file 4].

TNFα is a highly pleiotropic cytokine of inflammation that activates leukocytes and enhances adherence of neutrophils and monocytes to endothelium. As TNFα is synthesized by macrophages and other cells in response to pathogen-associated molecular patterns, inflammatory products, and other invasive stimuli, it mediates cellular responses through two distinct receptors, the p60 TNF receptor (TNFR1, p55) and the p80 TNF receptor (TNFR2, p75) [19]. These two receptors are present on the plasma membranes of virtually all cells except the erythrocyte, sharing structural homology in the extracellular TNFα-binding domains and exhibiting similar binding affinity for TNFα. However, they induce separate cytoplasmic signaling pathways following receptor-ligand binding. In order to investigate the TNF pathway clearly, TNFα-related proteins were extracted from the whole network, and the TNFα-related pathway is shown in Figure 5A. The inferred functional modules supported by literature evidences in the refined PPAN are further shown in Supplementary Table S2 [Additional file 3].

NFκB activation (TNFR1-TRADD-TRAF2-RIP) - One major capability of TNFα signaling pathway is of mediating the activation of inflammatory response transcription factor, NFκB. Upon activation of TNFR1 at the plasma membrane, the TNFR1 death domain (DD) serves as a docking site for the DD-containing adaptor protein TRADD through homotypic DD interactions [33]. TRADD then sequentially recruits TNF receptor-associated factor 2 (TRAF2) and the serine/threonine kinase RIP, which rapidly signals to NF-κB activation [33,34]. The functional module including proteins TNFR1, TRADD, TRAF2, and RIP were successfully identified in the PPAN.

NFκB activation (TNAF5-RIP, MEKK3-IKKβ-TAK1) - Two other functional modules which associated with NFκB activation were also recognized. TRAF5 has been implicated in the TNF-induced NF-κB activation, as in contrast to the single TRAF2 or TRAF5 knockout cells, TRAF2/TRAF5 double-knockout cells show impaired NF-κB activation upon TNF stimulation [35]. Unlike TRAF2, TRAF5 only interacts with RIP, but not with TRADD in co-immunoprecipitation assays [35], which can also been seen in the PPAN. In addition, MEKK3 has been shown to play a critical role in TNF-induced NF-κB activation using MEKK3-deficient fibroblast cells [36]. MEKK3 was also demonstrated to directly phosphorylate IKK and the kinase activity was regulated by TAK1 [36,37]. These functional interactions which result in NF-κB activation are observed in the refined PPAN.

IKK activation (RIP-IKKs) and protein recruitment (RIP-TAK1) - For the IKK activation, RIP responds to TNF and becomes K63-poly-ubiquitinated at lysine 377 in its intermediate domain. This is indispensable for IKK activation upon TNF stimulation, as mutation of lysine 377 abolishes the ability of RIP to rescue IKK activation in RIP-deficient cells [38]. RIP also plays a role in the recruitment of TAK1, as TAK1 fails to translocate to the TNFR1 complex upon TNF stimulation of RIP-deficient Jurkat cells [37].

Apoptosis (TRADD-RIP-FADD-TRAF2-CASP8) and protein kinase (TAK1-TAB1-TAB2) - Apoptosis is another critical function involved in the TNF pathway. TNFα binding will promote the complex forming of TNFR1 at the cell membrane. As TRADD, RIP and TRAF2 dissociate from TNFR1, and endosomal TNFR1 recruits the DD-containing adaptor protein FADD, which binds itself to caspase-8, a cytoplasmic complex forms and is implicated in signaling to apoptosis [39]. Moreover, the TAK1-TAB1-TAB2 complex is a protein kinase module which takes part in the TNFα signaling transduction and IKK activation. Activation of TAK1 leads to autophosphorylation of TAB1, whereas TAB2 becomes phosphorylated at the membrane, probably by an upstream protein kinase [40].

### Investigation of the IL-1R and TLR-4 signaling networks

The IL-1R/TLR superfamily can be clustered into multiple receptors, which all play a crucial role in both innate and adaptive inflammatory systems [12]. Members of the IL-1R subfamily are characterized by Ig-like domain that binds to specific IL-1-related cytokines, which is a primary regulator of inflammatory and inflammatory responses. By means of type I receptor (IL-1R1), it activates specific protein kinases, including the NFκB inducing kinase (NIK) and three distinct mitogen-activated protein (MAP) kinase cascades. These kinases modulate a number of transcription factors including NFκB, AP1 and CREB, each of which regulates a plethora expression of immediate early genes central to the inflammatory response [41]. On the other hand, the TLR subfamily includes 13 members that contain leucine-rich repeat motifs in their extracellular domains, which recognize distinct pathogen-associated patterns such as LPS, microbial lipopeptides, viral double-stranded RNA and CpG DNA [42]. These receptors have been established to play an essential role in the activation of inflammation and induce the release of critical pro-inflammatory cytokines that are necessary to activate potent inflammatory responses [43]. The cytoplasmic portions of both IL1R- and TLR-family members share a common structural motif, the so-called TLR and IL-1R (TIR) homology domain at their cytoplasmic portion [44]. Like TNFRs, TIR-containing receptors do not have catalytic activity and use intracellular adaptors and signal-transducing molecules to activate effector pathways [45]. Homotypic TIR-TIR interactions with a limited set of TIR-containing adaptors explain why more than 15 different receptors trigger only a small number of signaling pathways [45]. Because of the largely common use of signaling modules, the IL1R- and TLR4-related pathways were integrated into the same diagram for investigation (see Figure 5B). The inferred functional modules supported by literature evidences in the refined PPAN are further shown in Supplementary Table S3 [Additional file 4].

Pathway adaptor (MyD88-TLR4-IL1, IL1-IL1R1-MyD88-TOLLIP-IRAK1) - MyD88 is the universal adaptor for TLRs and is also a member of the IL-1 receptor subfamily [30]. Upon binding of IL-1, the IL-1R1 will associate with IL-1 receptor accessory protein (IL-1RAcP), forming a functional signaling receptor complex [28]. Then TIR domain containing adaptor protein MyD88 is recruited to the receptor complex [29]. This leads to the translocation of IRAK1, together with the adaptor protein TOLLIP [46], into the IL-1R1 and then IRAK1 interacts with TRAF6 [47]. The functional modules which are responsible for adaptation are observed in the refined PPAN.

Protein kinase (TRAF6-IRAK1-IRAK4, TRAF6-IRAK1-TAK1-TAB1-TAB2) and IKK activation (TRAF6-IKKs) - After forming the pathway adaptor modules, IRAK4 is recruited to TRAF6 and activated by intramolecular autophosphorylation [48]. Activation of IRAK4 leads to phosphorylation of IRAK1, procuring full kinase activity [48,49]. Then the IRAK-1-TRAF6 complex interacts with a pre-existing TAK1-TAB1-TAB2 membrane-bound complex [50], thus forming the protein kinase module. After that, the protein kinase module will translocate to the cytosol, whereas IRAK1 stays at the membrane and becomes polyubiquitinated. In the cytoplasm, TRAF6 interacts with the E2 ubiquitin-conjugating enzyme complex Ubc13/Uev1A [51]. In addition, lysine 124 in TRAF6 was identified as the main ubiquitin acceptor site for autoubiquitination, and mutation of this lysine leads to impaired TAK1, IKK and JNK activation [52,53]. Oligomerization of TRAF6 might lead to auto-polyubiquitination of TRAF6, which is necessary for IL-1-and LPS-induced NF-κB activation, whereas TRAF6- induced poly-ubiquitination of NEMO (NF-κB essential modulator) might rather play a role in IL-1-induced JNK activation [12].

### Cross-talk analysis of the refined PPANs

Our analyses have demonstrated many protein associations that showed characteristics of a real inflammatory system in the refined TNFα, IL-1, and TLR4 PPANs. To further understand the dynamic properties of hubs and cross-talks between different signaling pathways in the refined PPANs, we counted the CTRVs of each protein for cross-talk analysis of the refined PPANS. Proteins in this cross-talk analysis were classified into four major pathways, including TNFα, IL-1, MyD88-dependent and MyD88-independent pathways. In addition, negative regulators may also be important factors to be taken into consideration in this analysis because the cell responses must be stringently regulated. Exaggerated expression of signaling components and pro-inflammatory cytokines may cause devastating effects on the host. The negative regulators can act at multiple levels within inflammatory signaling cascades, as well as can elicit negative feedback mechanisms to synchronize the positive activation and negative regulation of signal transduction to avert potentially harmful consequences [23]. The results of the cross-talk analysis are shown in Table 3 and Table 4. In Table 3, the CTRVs and link values, which indicated the total numbers of association for each node at six different time stages, were listed for all 60 proteins. The contrast between link and CTRVs (for example, CASP8) reflected that not all proteins with high connective degree in the refined PPAN would also be with high CTRVs. In Table 4, proteins with high CTRVs at different time stages are presented. From Table 2 and Table 4, we found that some proteins with high CTRVs at different time stages such as NIK and A20 were not ranked top according to node degree, revealing that our proposed method generated new insights into the evaluation of cross-talk candidates.

**Table 3:** Cross-talk ranking values (CTRVs) and link values of significant proteins in PPAN.

**No**.	**Protein**	**CTRV**	**Link**	**No**.	**Protein**	**CTRV**	**Link**
	
1	IRAK1	108	140	31	IL1R1	21	21
2	TRAF6	83	124	32	FADD	19	55
3	NIK	77	89	33	GRB2	18	46
4	A20	61	61	34	TAK1	17	66
5	MYD88	59	107	35	SOCS1	17	17
6	TRAF2	58	198	36	TRIF	16	54
7	IKKα	55	83	37	TMED1	12	12
8	SIGIRR	53	73	38	CASP8	10	87
9	TLR4	53	53	39	TAB2	10	53
10	IKKγ	52	84	40	CAV1	10	44
11	IKKβ	47	97	41	TAB1	9	44
12	BCL10	43	62	42	TTRAP	8	8
13	UBE2N	41	41	43	ECSIT	8	50
14	IRAK4	38	112	44	CYLD	6	6
15	RIP	38	57	45	TNF	0	48
16	TRAF3	37	57	46	TNFR2	0	39
17	IRAK3	36	36	47	IKKε	0	29
18	ST2L	32	32	48	PELI2	0	19
19	TNIP3	32	32	49	IL1B	0	18
20	PTPN11	31	31	50	MEKK3	0	18
21	TIRAP	29	40	51	TRAM	0	18
22	TRAF4	27	27	52	IL1A	0	17
23	TOLLIP	26	47	53	IL1R2	0	17
24	RNF216	25	25	54	TRAF5	0	17
25	TNFR1	24	111	55	SOS1	0	10
26	RIP3	23	93	56	IRF3	0	9
27	TRADD	23	32	57	NOD2	0	0
28	TBK1	22	39	58	CD14	0	0
29	PELI1	21	49	59	SARM1	0	0
30	TANK	21	40	60	FLN29	0	0

To integrate the information from the complex PPANs, the cross-talk ranking values (CTRVs) at different time stages are summed up for each protein. In contrast with CTRVs, the values of link represent the total number of protein-protein associations at six time stages of each protein. It reveals that not all nodes with high connective degree will also have high CTRVs such as CASP8.

**Table 4:** Proteins with high cross-talk ranking values at different time stages.

Duration	Top ranked proteins				
**0~1HR**	IRAK1	NIK	IKKα	TRAF6	IKKβ
**1~2HR**	TRAF6	IRAK1	NIK	A20	TRAF2
**2~3HR**	IRAK1	TRAF6	NIK	A20	TRAF2
**3~4HR**	IRAK1	NIK	TRAF6	A20	IKKγ
**4~6HR**	IRAK1	TRAF6	NIK	TRAF2	A20
**6~8HR**	IRAK1	NIK	TRAF6	IKKα	A20

In contract with Table 2, proteins with maximal CTRVs are listed here. Among these highly ranked proteins, NIK and A20 are considered as two core elements in the signaling network by the analysis of CTRV, but their importance is not exhibited in Table 2. This reveals that our proposed method generates a new insight into the evaluation of cross-talk candidates.

The biological significance of some highly ranked proteins we identified (Table 3) was investigated. Interleukin-1 receptor-activated kinase 1 (IRAK1, ranked No.1) is one of the key mediators in the signaling pathways of TLRs/IL-1Rs. IRAKs which initiate a cascade of signaling events eventually lead to induction of inflammatory target gene expression [54]. IRAK1 activation constitutes an important signaling module in both IL-1R and TLR signal transductions. Binding to myeloid differentiation primary response protein (MyD88, ranked No.5) brings IRAK1 and IRAK4 together at the receptor complex and facilitates the phosphorylation of IRAK1. In the downstream of MyD88, the important role of IRAK and TRAF6 in related pathways has also been confirmed in some knockout studies. Cells from IRAK-deficient mice were shown to be defective in their response to IL-1 and IL-18 [55]. Tumor necrosis factor receptor-associated factor 6 (TRAF6, ranked No.2) is a pivot signaling molecule regulating a diverse array of physiological processes including adaptive immunity, innate immunity and the development of several tissues. It is also essential for the signaling downstream of the IL-1R/TLR superfamily [56]. The crucial biological role of TRAF6 in the IL-1R/TLR signaling has been demonstrated by the targeted deletion of TRAF6 [57,58]. Mitogen-activated protein kinase kinase kinase 14 (NIK, ranked No.3) is a member of the MAPKKK family that may either directly or indirectly phosphorylate or activate IKKα/β, leading to the phosphorylation and degradation of IκBα followed by NFκB activation [59]. NIK is also a common mediator of NF-κB activation by the TNF receptor family and shown to activate the downstream of TRAF-associating receptor signaling pathways, including TNFR, CD40, CD30 and LTβr [60,61]. Tumor necrosis factor, alpha-induced protein 3 (TNFAIP3/A20, ranked No.4) is a protein which can be induced in many cell types and by a wide range of stimuli [62]. Although A20 was originally characterized as an inhibitor of TNF-induced apoptosis [63], it has been most intensively studied as an inhibitor of NF-κB activation. The use of A20-deficient mice and RNA interference technologies has revealed the crucial role of A20 in a variety of pathogen- and cytokine-induced signaling pathways. Mice lacking A20 are born at normal Mendelian ratios but die shortly after birth due to massive multi-organ inflammation, which is an indication of a key role for A20 in homeostasis of the host [64].

Although there are still many highly ranked proteins with critical roles in signal transduction like MyD88 and SIGIRR, it is not our intension to use an exhaustive attack method to prove them one by one. Instead, the global properties and robustness of the network architecture in the sequel will be investigated.

## Discussion

In this study, the rough PPANs are constructed at first based on the selected proteins of interest and the database information of protein-protein interactions. We then use equation 1 to mathematically describe the relationship between the target protein and the proteins possibly interacting with it in the rough PPAN. For each protein in the rough PPAN, the possible interactions are established via equation 1. Next, with the help of microarray data, the parameters in equation 1 are identified using the constrained least squares estimation method, i.e. every interaction should be confirmed by the real microarray data. Finally, the Akaike Information Criterion (AIC) is used to determine the insignificant *b_pq_*'s in equation 1, thus pruning the initial rough PPAN into the refined PPAN by detecting the insignificant interactions. The data set of 25 time points we used is divided into six time stages, and six refined PPANs are constructed based on these six subsets of data. Some dynamic characteristics and structures of the reconstructed PPANs of inflammation are discussed in the following.

### Dynamic progression of the PPANs

**Figure 6:**
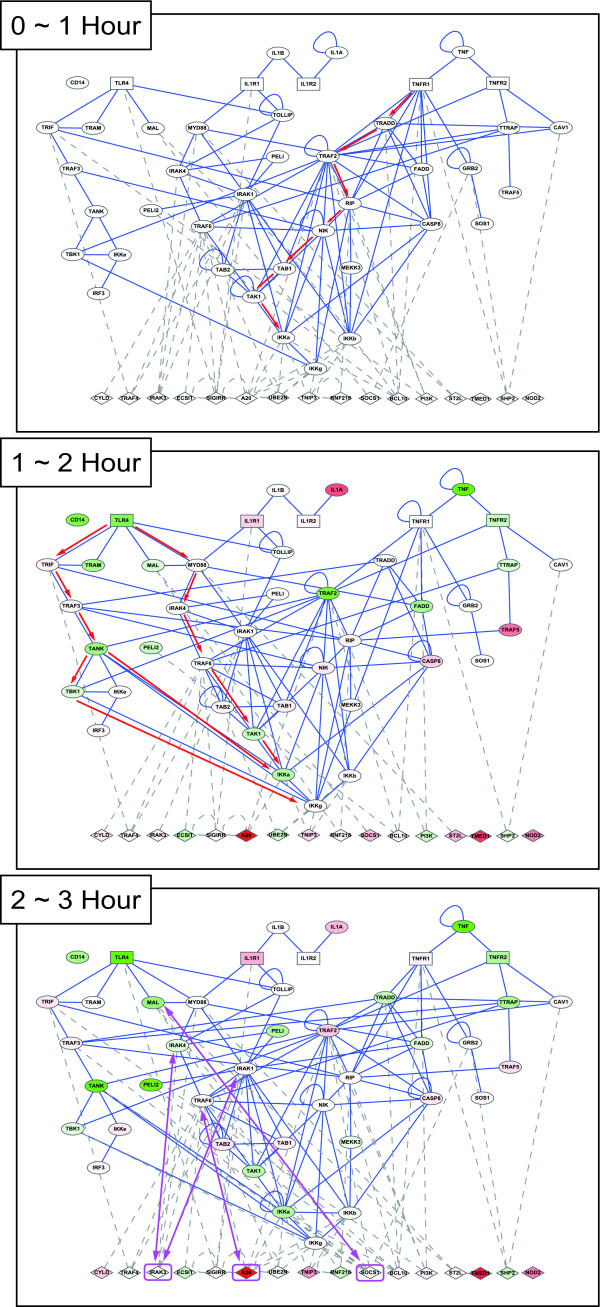
**Dynamic progression of refined PPANs for HUVEC under TNFα stress**. Time series refined PPANs under the TNFα stress from 0 to 3 hours are presented to monitor the dynamic properties of association progression. Positions of nodes are rearranged based on approximately up/down-stream relationships of the proteins. The general signaling proteins are shown as elliptic nodes; square nodes represent the receptor proteins and diamond nodes represent the possible negative regulator proteins. Dash gray lines represent the associations which are related to negative regulators. The levels of gene expression are indicated by the node color, in which the red color means the gene expression at that time is higher than its gene expression without TNFα treatment and the green color means the gene expression at that time is lower than its gene expression without TNFα treatment. The complete and pellucid time series diagrams from 0 to 8 hours are presented in Supplementary Figures [Additional file 5].

To determine the dynamic progression of PPANs at different time stages, we presented the time series PPANs from 0 to 3 hours in which the positions of nodes are rearranged based on approximately up/down-stream relations (see Figure 6, and the complete and pellucid time series diagrams from 0 to 8 hours are presented in Supplementary Figures [Additional file 5]).

In the first hour, there is a very obvious signal cascade which passes through TNFR1, TRADD, TRAF2, RIP, NIK, TAK1/TAB1/TAB2 complex and finally reaches the IKK complex (see Figure 6, 0~1 hour, red edges). It seems to be the rapidest way to activate the NF-κB transcription by TNFα induction. This well-characterized pathway contains NF-κB, JNK, p42/p44 mitogen-activated protein kinase (MAPK) and p38 MAPK [19]. Interestingly, NIK, which seems to be an important mediator between RIP and IKK family in our network diagram, was not previously considered to be a part of the TNF-induced NF-κB activation. However, Yin *et al. *found that NIK is required for NF-κB activation by LTβR [65]. In addition, MAP/ERK kinase kinase kinase 3 (MEKK3) is also involved in this pathway by RIP. Gene deletion studies have indicated that MEKK3 is required for IKK activation and functions downstream of RIP in TNF-induced NF-κB activation [66]. In the second hour, the IL-1R/TLR4 signaling pathways are sequentially turned on after the TNFR-related pathway (see Figure 6, 1~2 hour, red edges). This observation suggests the movement of autocrine signaling, in which a cell secretes a hormone or chemical messenger that binds to autocrine receptors on the same cell type, leading to changes in the cells. At this time point, the connections in the TNFR pathway become relatively less than those shown in the first hour and even display an inhibitory effect on this pathway. The reason for the rearrangement of protein associations might be that the cell's inflammatory mechanism tends to focus on some specific mechanisms which largely share common community to fight against the pathogens, rather than distributes resources to the overextended pathways. In the third hour, the TNFR-related pathway is triggered again. It is noticed that several negative regulators such as SOC1 (inhibitor of TIRAP (MAL) and p56), IRAK3 (inhibitor of IRAK1 and IRAK4) and A20 (inhibitor of TRAF6) are significantly expressed at this stage, reflecting the inhibitory effect of anti-inflammation (see Figure 6, 2~3 hour, purple edges). Apart from these three refined PPANs, the dynamic properties of another three residual networks of the late stages of immunity (3 to 4, 4 to 6 and 6 to 8 hours) are more like at steady state without significant perturbations (see Supplementary Figures [Additional file 5]).

### Specific architecture in the signaling network

**Figure 7:**
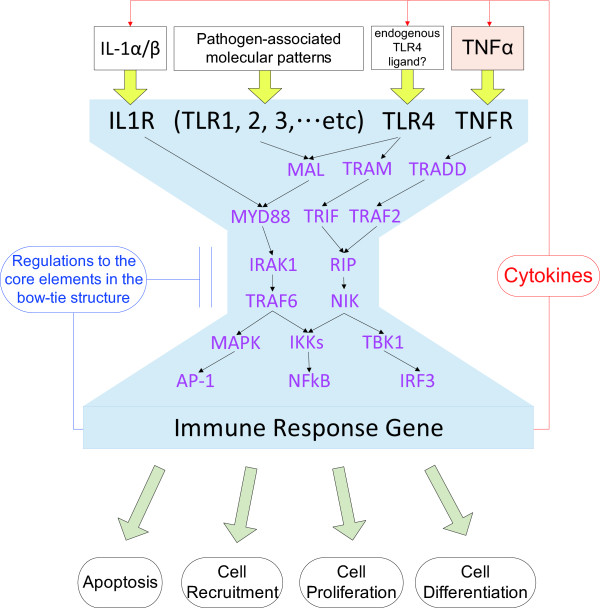
**Bow-tie structure under TNFα stress for multiple pathways in the inflammatory system**. A specific architecture of TNFα-induced endothelial inflammatory system is extracted from the PPAN in which the core elements of the bow-tie structure are identified via the CTRV ranking algorithm. Upon ligand binding, various types of receptors on the cell membrane trigger different signaling pathways and activate the downstream corresponding transcription factors such as NFκB. NFκB then regulates the expression of genes involved in inflammatory responses. These kinds of gene expression will induce some particular biological mechanisms helping the host to defense the invading microorganisms. In addition, the translation of cytokines and some negatively regulatory proteins will play roles of feedback control to coordinate the balance in immunity.

A cell's behavior is a consequence of the complex interactions between its numerous constituents, such as DNA, RNA, proteins and small molecules. Cells use signaling pathways and regulatory mechanisms to coordinate multiple processes, allowing them to respond and adapt to an ever-changing environment. In case of pathogen invading, the human inflammatory system is required to rapidly take appropriate response to eliminate or moderate the lethal factors without unnecessary wastage. Figure 7 displays the bow-tie structure extracted from the PPANs. The core elements in the bow-tie structure are the highly ranked proteins according to the CTRV ranking algorithm. As different receptors on the cell membrane are only able to recognize their specific pathogen-associated molecular patterns (PAMPs), these receptors need to form various functional modules by complex and dimer/trimer assembling for representing different signals of pathogens. Then these various types of signal would converge to a common cross-talk, that is, the core elements in the bow-tie structure. Therefore, the cross-talks may be considered as a robust and efficient signal processor on the pivotal position of signal transduction, which plays a role of rearranging the sinew and determines which necessary protective mechanisms or recruitments should be activated. Through the coordination of signal processor, inflammatory system organizes the order and balance in the cell and human body.

Oda and Kitano manually integrated 411 published literatures and presented a comprehensive map of TLRs and IL-1 receptor signaling networks under different stimulant conditions [67]. This map illustrates the possible existence of a main network subsystem that has a bow-tie structure in which MyD88 is a non-redundant core element, two collateral subsystems with small GTPase and phosphatidylinositol signaling, and MyD88-independent pathway [67]. In comparison of our ranking results with their signaling map, it reveals that the top ranked proteins such as IRAK1 (ranked No.1), TRAF6 (ranked No.2) and MyD88 (ranked No.5) in our study have also been considered the pivotal roles in the bow-tie core process. Specifically, the process mediates various types of stimulant signals and triggers the downstream activation of NF-κB and MAPK cascade, leading to the induction of many target genes such as cytokines. However, the map of Oda and Kitano only reflects the possible static connections without stimulus-specific response or temporal changes. In contrast, we integrated the gene expression patterns from time course data to infer the dynamic protein-protein interactions and networks. Consequently, our results may suggest a more significant and realistic bow-tie core network under a specific stimulus.

### Possible existence of TLR4 endogenous ligand

Though the main focus of our study is the TNFRs that are different from the TLRs in Oda and Kitano [67], numerous similar functional modules are identified in this study. This observation reveals the characteristics of module community in the inflammatory system and the presence of active feedback signals from cytokines. It is proven that TNFα-induced cells will activate the transcriptional expression of several genes encode cytokines such as IL-1α, IL-1β and IL-6 [27]. These autocrine signals can act as the positive feedback to enhance the inflammatory responses by turning on other correlated inflammatory pathways, such as IL-1 and MyD88-dependent pathway of TLR4. Interestingly, in addition to the two pathways mentioned above, our TNFα-induced HUVEC model also exhibits the virtually complete activation of MyD88-independent pathway of TLR4 which theoretically may not be involved in the single TNFα-treated condition (see Figure 5B). It has been shown that increased expression and signaling by TLR4 may contribute to the activation of innate immunity in the injured myocardium [68]. Because no infection is evident in this model, our observation raises the intriguing possibility that TLR4 may also function during inflammation, possibly in response to an endogenous ligand. One candidate of this ligand is S100, a multigenic family of non-ubiquitous Ca^2+^-modulated proteins of the EF-hand type expressed in vertebrates exclusively [69]. It has been demonstrated that primary tumors secrete soluble factors, including VEGF-A, TGFβ and TNFα, which induce expression of S100 in the myeloid and endothelial cells within the lung prior to tumor metastasis [70]. Recently, the increased S100A8 and S100A9 levels were also detected in various human cancers, presenting abundant expression in neoplastic tumor cells as well as infiltrating immune cells [71]. Its expression and potential cytokine-like function in inflammation and in cancer suggests that S100A8/A9 may play a key role in inflammation-associated cancer. Another candidate is high-mobility group box 1 (HMGB1), which is one kind of damage-associated molecular patterns (DAMPs). HMGB1 is a nuclear protein expressed in nearly all cell types. In normal conditions, HMGB1 binds to DNA and facilitates gene transcription. Under stress conditions such as injury and infection, HMGB1 is released and promotes inflammation [72]. TLR4 has been identified as a receptor of HMGB1 as well as TLR2 and RAGE (receptor of advance glycation end product) [73]. As the mechanisms of promoting the release of HMGB1 and its activating signaling pathways remain to be completely elucidated, HMGB1 also appears to have several regenerative effects leading to tissue repair [72,74]. Therefore, HMGB1 has potential significance in clinical medicine.

### Negative feedback controls of the cross-talks

Inflammation is normally a protective response to destroy, dilute, or isolate an eliciting agent and to promote the repair of injured tissue. However, when inflammation is excessive or persistent, it may cause tissue injury or organ dysfunction and may contribute to the pathogenesis of disease. For this reason, at the anti-inflammation stage cells need to make use of the negative regulator proteins and cytokines to inhibit the function of some dominant cross-talks to recover from the dramatic activations in inflammation. This kind of negative feedback has also been proven to be related to some TNFα-mediated inflammatory diseases. For example, A20 has been identified as a negative regulator of the core cross-talk element TRAF6 [75], and several studies have shown that deficiencies in A20 are associated with some autoimmune diseases, including rheumatoid arthritis [76], systemic lupus erythematosus [77], and Crohn's disease [78]. These clues also reveal the fragility of the bow-tie structure. Because of the non-redundant property of core elements in the bow-tie structure, some targeted perturbations from environment and lethal pathogens might result in destructive consequences.

On the other hand, negative feedback controls may also play an important role to buffer the wide range of environment stimuli. Previous studies have indicated that proteasome inhibition can suppress TNFα-induced activation of adhesion molecules in endothelial cells *in vitro *[79,80]. These studies showed that short-term treatment of endothelial cells with high doses of proteasome inhibitors results in strong inhibition of cytokine-induced expression by preventing nuclear translocation of NF-κB. Cheong *et al. *undertook an iterative computational and experimental investigation of the dynamic properties of TNFα-mediated activation of the transcription factor NF-κB [81]. They found that the temporal profile of the NF-κB activity is invariant to the TNFα dose from 0.1 to 10 ng/ml. These discoveries reflect the properties of robustness and protective mechanisms in the inflammatory system which might also be the effects of feedback controls to the cross-talks in the signaling network. As the environmental stresses trigger the appropriate responses to protect organisms themselves, it will help their survival. However, if the stresses suddenly perturb to an acute level and the inflammatory responses still take a correspondingly sharp response to excessively activate the downstream reactions, the protective mechanisms will instead injure the organisms. For this reason, there must be some cross-talks which buffer the perturbation from upstream signals and respond to the downstream negative feedback regulators to alleviate the reflecting scale.

## Conclusions

In this study we attempt to integrate protein-protein interactions from databases and gene expression profiles of TNFα-induced HUVEC to construct the protein-protein association networks (PPANs) at different time stages to illustrate the development of an endothelial inflammatory system. A new cross-talk ranking method is suggested to evaluate the potential core elements in the related signaling pathways of toll-like receptor 4 (TLR-4) as well as receptors for tumor necrosis factor (TNF-R) and interleukin-1 (IL-1R). The highly ranked cross-talks which are functionally relevant to the TNFα stress are also identified. A bow-tie structure is then extracted from these cross-talk pathways for the robustness of network structure, the coordination of signal transduction and the feedback control for efficient inflammatory responses to different stimuli. Further, several characteristics such as possible existence of TLR4 endogenous ligand and the effects of negative feedback control to the cross-talks are discussed.

A systematic approach based on stochastic dynamic model is proposed for biologists to generate insight into the underlying defense mechanisms of endothelial inflammatory systems via the construction of corresponding signaling networks upon specific stimulus. The dynamic model provides a mathematical regulatory mechanism of the biological networks, resulting in not only qualitative but also quantitative dynamic characterization. This model can also be integrated with the downstream signaling networks such as PPAN of NFκB and gene regulatory networks [82] to investigate more comprehensive issues. Further, based on the dynamic model, we can also discuss the robust stability and noise filtering ability of the biological network against the intrinsic fluctuation and environmental disturbances [83]. In addition, this systematic approach can be applied to other signaling networks under different conditions in different species. As better experimental techniques for protein expression detection and microarray data with multiple sampling points become available in the future, the performance of the proposed method will be much improved and the dynamic PPANs under different conditions can be compared extensively.

## Competing interests

The authors declare that they have no competing interests.

## Authors' contributions

SKY developed the method, performed the simulation, evaluated the results and drafted the manuscript. YCW developed the method, helped to perform the simulation and drafted the manuscript. CCC participated in the method development and result evaluation. YJC and CYL provided essential guidance and revised the manuscript. BSC conceived of the study and revised the manuscript. All authors read and approved the final manuscript.

## Pre-publication history

The pre-publication history for this paper can be accessed here:

http://www.biomedcentral.com/1755-8794/3/19/prepub

## Supplementary Material

Additional file 1 **Supplementary Table S1**. The Gene Ontology annotations (biological process, molecular function, cellular component) for the 60 proteins of interest are shown in the xls file. Click here for file

Additional file 2 Supplementary Methods. Click here for file

Additional file 3 **Supplementary Table S2**. Investigation of the TNFα refined protein-protein association network Click here for file

Additional file 4 **Supplementary Table S3**. Investigation of the IL-1 and TLR4 refined protein-protein association network. Click here for file

Additional file 5 **Supplementary Figures**. Complete time series diagrams of the refined PPANs under TNFα stress from 0 to 8 hour are shown in Supplementary Figures. Click here for file
