# Supplementary material for: Dynamic cross-talk analysis among TNF-R, TLR-4 and IL-1R signalings in TNFα-induced inflammatory responses
Source: BMC Med Genomics. 2010 May 24;3:19. doi: 10.1186/1755-8794-3-19 (PMC2889840; doi:10.1186/1755-8794-3-19)
Supplement: Additional file 2 — Supplementary Methods. [file 1755-8794-3-19-S2.PDF]

Supplementary Methods

Identification of the regulatory parameters

After constructing the stochastic dynamic model of the rough PPANs from equation (1), we have to identify the regulatory parameters in the model with the gene expression signatures. Since the parameters in equation (1) have certain constraints ($\alpha_p, \beta_p \geq 0$), we identify the regulatory parameters by solving the constrained least squares problems.

Equation (1) can be rewritten in the following regression form

$$y_p[t+1] = \begin{bmatrix} y_p[t] & y_p[t]y_1[t] & \cdots & y_p[t]y_Q[t] & x_p[t] & -y_p[t] \end{bmatrix} \cdot \begin{bmatrix} 1 \\ b_{p1} \\ \vdots \\ b_{pQ} \\ \alpha_p \\ \beta_p \end{bmatrix} + \omega_p[t] \quad \text{(S1)}$$

$$\equiv \psi_p[t] \cdot \eta_p + \omega_p[t]$$

where $\psi_p[t]$ indicates the regression vector and η_p is the parameter vector to be estimated. By the cubic spline method, at different time points, equation (S1) can be presented as the following equation [1].

$$Y_n = \Psi_n \cdot \eta_n + \Omega_n \quad \text{(S2)}$$

The identification problem is then formulated as follows

$$\min_{\eta_n} \frac{1}{2} \left\| \Psi_n \eta_n - Y_n \right\|_2^2 \quad \text{such that } C\eta_n \leq d \quad \text{(S3)}$$

where $C = \text{diag}\begin{bmatrix} 0 & \cdots & 0 & -1 & -1 \end{bmatrix}$, $d = \begin{bmatrix} 0 & \cdots & 0 & 0 \end{bmatrix}^T$ give the constraints to force the translation effect α_p and the degradation effect β_p in equation (1) to be always non-negative, i.e., $\alpha_p, \beta_p \geq 0$. The constrained least squares problem can be

solved using the active set method for quadratic programming.

Here the gene expression profiles were adapted to infer the dynamic changes of associations at different time stages, because we do not have trustworthy protein microarray which can measure thousands of protein expression levels simultaneously as DNA microarray. If the experimental techniques are developed to offer the high-throughput protein expression profiles, identification of the regulatory parameters can be more reliable. In addition, due to the lack of protein expression data and the non-direction of the protein interactions in the rough PPAN, the values of the association parameters b_{pq}'s are not deeply discussed in this study. Instead, we take a system view to investigate the global properties and the network development at serial time stages.

Determination of significant interaction pairs

After identifying the parameters of the dynamic model in equation (1), there are still some insignificant association coefficients being identified. In order to determine whether a regulatory protein is significant or not in the rough PPAN, a statistical approach based on model selection is proposed for evaluating the significance of our model parameters to prune the rough PPAN. We employ Akaike Information Criterion (AIC) [2, 3] for determination of significant interactions in the rough PPAN. AIC is a model selection method which attempts to include both the estimated residual variance and model complexity in one statistic. It decreases as the residual variance decreases and increases as the number of parameters increases. As the expected residual variance decreases with increasing parameter numbers for non-adequate model complexities, there should be a minimum near the correct parameter number [2, 3]. Therefore, we can use AIC to select model structure based on the association parameters (b_{pq}'s) identified above.

References

1. Wang YC, Chen BS: **Integrated cellular network of transcription regulations and protein-protein interactions.** *BMC Systems Biology* 2010, **4:**20.
2. Akaike H: **A new look at the statistical model identification.** *Automatic Control, IEEE Transactions on* 1974, **19:**716-723.
3. Johansson R: *System modeling and identification.* Englewood Cliffs, NJ: Prentice Hall; 1993.
