# Supplementary material for: Dynamic cross-talk analysis among TNF-R, TLR-4 and IL-1R signalings in TNFα-induced inflammatory responses
Source: BMC Med Genomics. 2010 May 24;3:19. doi: 10.1186/1755-8794-3-19 (PMC2889840; doi:10.1186/1755-8794-3-19)
Supplement: Additional file 3 — Supplementary Table S2. Investigation of the TNFα refined protein-protein association network [file 1755-8794-3-19-S3.PDF]

Supplementary Table S2

Investigation of the TNFα refined protein-protein association network

Function	Related proteins	Modules extracted from PPANs	Evidence
NFκB Activation	**TNFR1**<br>**TRADD**<br>**TRAF2**<br>**RIP**	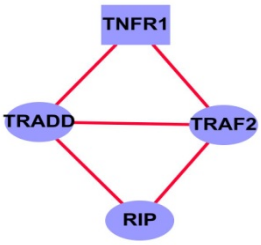	[1, 2]
	**TRAF5**<br>**RIP**	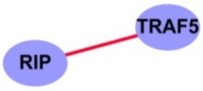	[3]
	**MEKK3**<br>**IKKβ**<br>**TAK1**	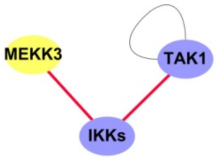	[4, 5]
IKK Activation	**RIP**<br>**IKKs**	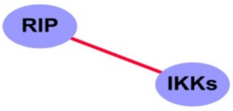	[6]
Apoptosis	**TRADD**<br>**RIP**<br>**FADD**<br>**TRAF2**<br>**CASP8**	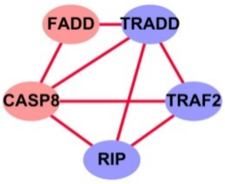	[7]
Protein Recruitment	**RIP**<br>**TAK1**	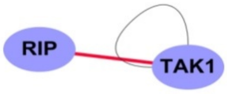	[5]
Protein Kinase	**TAK1**<br>**TAB1**<br>**TAB2**	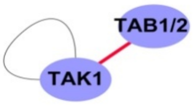	[8]

Modules with significant protein-protein associations which form some specific functional complex are extracted from Figure 4A. The references which support these associations are listed in the evidence column, and the effects of these functional associations are described in the text.

References

1. Hsu H, Shu HB, Pan MG, Goeddel DV: **TRADD-TRAF2 and TRADD-FADD interactions define two distinct TNF receptor 1 signal transduction pathways.** *Cell* 1996, **84:**299-308.
2. Legler DF, Micheau O, Doucey MA, Tschopp J, Bron C: **Recruitment of TNF receptor 1 to lipid rafts is essential for TNFalpha-mediated NF-kappaB activation.** *Immunity* 2003, **18:**655-664.
3. Tada K, Okazaki T, Sakon S, Kobarai T, Kurosawa K, Yamaoka S, Hashimoto H, Mak TW, Yagita H, Okumura K, et al: **Critical roles of TRAF2 and TRAF5 in tumor necrosis factor-induced NF-kappa B activation and protection from cell death.** *J Biol Chem* 2001, **276:**36530-36534.
4. Yang J, Lin Y, Guo Z, Cheng J, Huang J, Deng L, Liao W, Chen Z, Liu Z, Su B: **The essential role of MEKK3 in TNF-induced NF-kappaB activation.** *Nat Immunol* 2001, **2:**620-624.
5. Blonska M, Shambharkar PB, Kobayashi M, Zhang D, Sakurai H, Su B, Lin X: **TAK1 is recruited to the tumor necrosis factor-alpha (TNF-alpha) receptor 1 complex in a receptor-interacting protein (RIP)-dependent manner and cooperates with MEKK3 leading to NF-kappaB activation.** *J Biol Chem* 2005, **280:**43056-43063.
6. Ea CK, Deng L, Xia ZP, Pineda G, Chen ZJ: **Activation of IKK by TNFalpha requires site-specific ubiquitination of RIP1 and polyubiquitin binding by NEMO.** *Mol Cell* 2006, **22:**245-257.
7. Hsu H, Huang J, Shu HB, Baichwal V, Goeddel DV: **TNF-dependent recruitment of the protein kinase RIP to the TNF receptor-1 signaling complex.** *Immunity* 1996, **4:**387-396.
8. Wang C, Deng L, Hong M, Akkaraju GR, Inoue J, Chen ZJ: **TAK1 is a ubiquitin-dependent kinase of MKK and IKK.** *Nature* 2001, **412:**346-351.
