# Supplementary material for: Dynamic cross-talk analysis among TNF-R, TLR-4 and IL-1R signalings in TNFα-induced inflammatory responses
Source: BMC Med Genomics. 2010 May 24;3:19. doi: 10.1186/1755-8794-3-19 (PMC2889840; doi:10.1186/1755-8794-3-19)
Supplement: Additional file 4 — Supplementary Table S3. Investigation of the IL-1 and TLR4 refined protein-protein association network. [file 1755-8794-3-19-S4.PDF]

Supplementary Table S3

Investigation of the IL-1 and TLR4 refined protein-protein association network

<table>
<tr><th>Function</th><th>Related proteins</th><th>Modules extracted from PPANs</th><th>Evidence</th></tr>
<tr><td rowspan="2">Pathway Adaptor</td><td>MyD88
TLR4
IL1R1</td><td>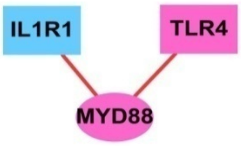
</td><td>[1]</td></tr>
<tr><td>IL1
IL1RI
MyD88
TOLLIP
IRAK1</td><td>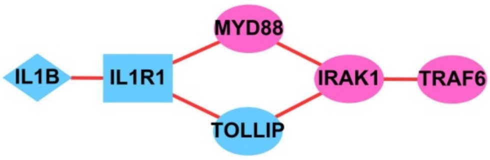
</td><td>[2-5]</td></tr>
<tr><td rowspan="2">Protein Kinase</td><td>TRAF6
IRAK1
IRAK4</td><td>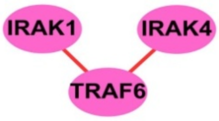
</td><td>[6, 7]</td></tr>
<tr><td>TRAF6
IRAK1
TAK1
TAB1
TAB2</td><td>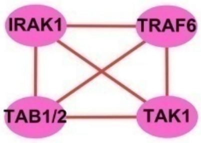
</td><td>[8-11]</td></tr>
<tr><td>IKK Activation</td><td>TRAF6
IKKs</td><td>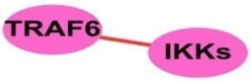
</td><td>[12]</td></tr>
</table>

Modules with significant protein-protein associations which form some specific functional complex are extracted from Figure 4B. The references which support these associations are listed in the evidence column, and the effects of these functional associations are described in the text.

References

1. Medzhitov R, Preston-Hurlburt P, Kopp E, Stadlen A, Chen C, Ghosh S, Janeway CA, Jr.: **MyD88 is an adaptor protein in the hToll/IL-1 receptor family signaling pathways.** *Mol Cell* 1998, **2:**253-258.
2. Greenfeder SA, Nunes P, Kwee L, Labow M, Chizzonite RA, Ju G: **Molecular cloning and characterization of a second subunit of the interleukin 1 receptor complex.** *J Biol Chem* 1995, **270:**13757-13765.
3. Wesche H, Henzel WJ, Shillinglaw W, Li S, Cao Z: **MyD88: an adapter that recruits IRAK to the IL-1 receptor complex.** *Immunity* 1997, **7:**837-847.
4. Zhang G, Ghosh S: **Negative regulation of toll-like receptor-mediated signaling by Tollip.** *J Biol Chem* 2002, **277:**7059-7065.
5. Ye H, Arron JR, Lamothe B, Cirilli M, Kobayashi T, Shevde NK, Segal D, Dzivenu OK, Vologodskaia M, Yim M, et al: **Distinct molecular mechanism for initiating TRAF6 signalling.** *Nature* 2002, **418:**443-447.
6. Cheng H, Addona T, Keshishian H, Dahlstrand E, Lu C, Dorsch M, Li Z, Wang A, Ocain TD, Li P, et al: **Regulation of IRAK-4 kinase activity via autophosphorylation within its activation loop.** *Biochem Biophys Res Commun* 2007, **352:**609-616.
7. Li X, Commane M, Burns C, Vithalani K, Cao Z, Stark GR: **Mutant cells that do not respond to interleukin-1 (IL-1) reveal a novel role for IL-1 receptor-associated kinase.** *Mol Cell Biol* 1999, **19:**4643-4652.
8. Jiang Z, Ninomiya-Tsuji J, Qian Y, Matsumoto K, Li X: **Interleukin-1 (IL-1) receptor-associated kinase-dependent IL-1-induced signaling complexes phosphorylate TAK1 and TAB2 at the plasma membrane and activate TAK1 in the cytosol.** *Mol Cell Biol* 2002, **22:**7158-7167.
9. Lamothe B, Besse A, Campos AD, Webster WK, Wu H, Darnay BG: **Site-specific Lys-63-linked tumor necrosis factor receptor-associated factor 6 auto-ubiquitination is a critical determinant of I kappa B kinase activation.** *J Biol Chem* 2007, **282:**4102-4112.
10. Sato S, Sanjo H, Takeda K, Ninomiya-Tsuji J, Yamamoto M, Kawai T, Matsumoto K, Takeuchi O, Akira S: **Essential function for the kinase TAK1 in innate and adaptive immune responses.** *Nat Immunol* 2005, **6:**1087-1095.
11. Yang K, Zhu J, Sun S, Tang Y, Zhang B, Diao L, Wang C: **The coiled-coil domain of TRAF6 is essential for its auto-ubiquitination.** *Biochem Biophys Res Commun* 2004, **324:**432-439.
12. Verstrepen L, Bekaert T, Chau TL, Tavernier J, Chariot A, Beyaert R: **TLR-4, IL-1R and TNF-R signaling to NF-kappaB: variations on a common theme.** *Cell Mol Life Sci* 2008, **65:**2964-2978.
