# Supplementary figures and images for: Dynamic cross-talk analysis among TNF-R, TLR-4 and IL-1R signalings in TNFα-induced inflammatory responses
Source: BMC Med Genomics. 2010 May 24;3:19. doi: 10.1186/1755-8794-3-19 (PMC2889840; doi:10.1186/1755-8794-3-19)

0 ~ 1 Hour

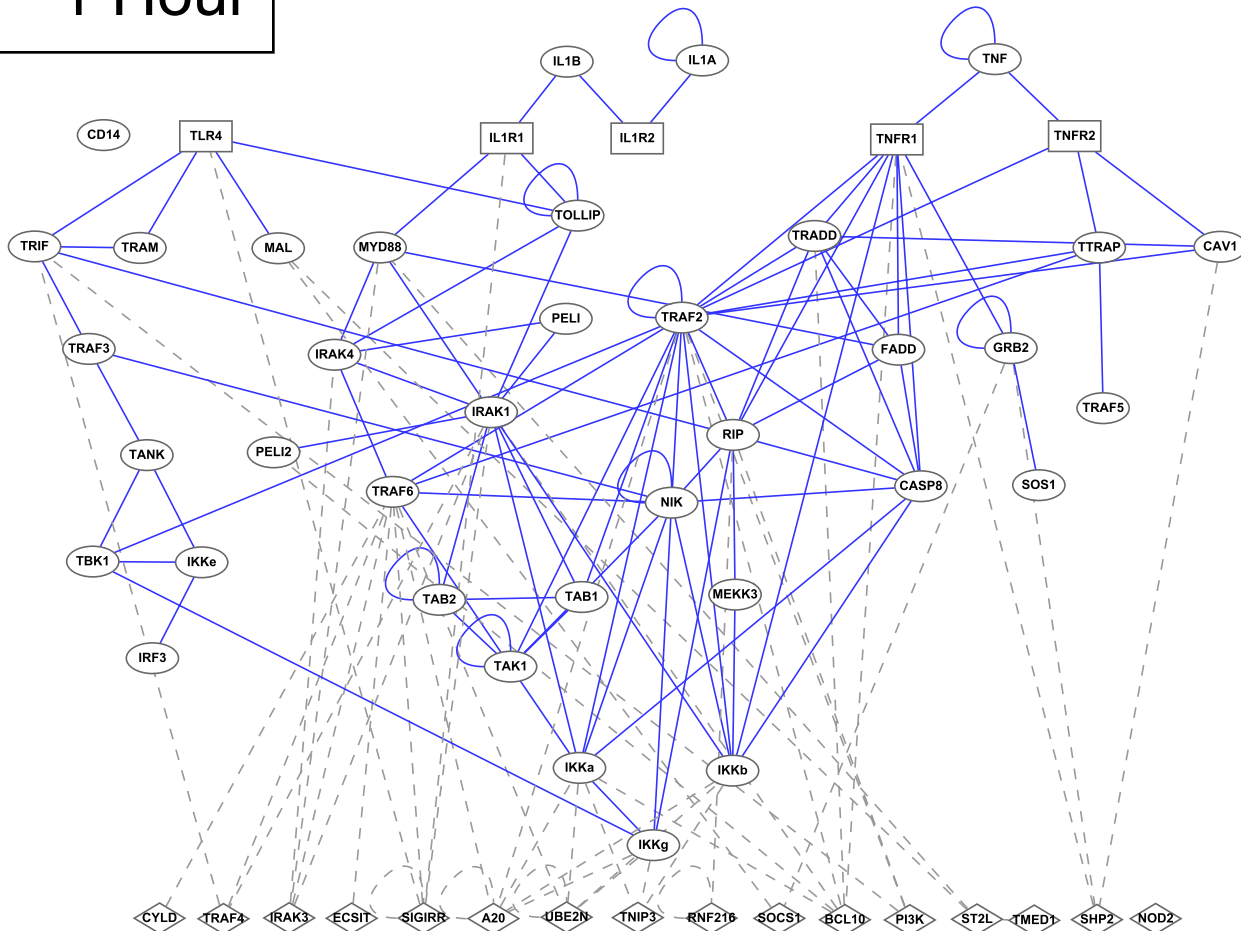

Supplement: Additional file 5 — Supplementary Figures. Complete time series diagrams of the refined PPANs under TNFα stress from 0 to 8 hour are shown in Supplementary Figures. [file 1755-8794-3-19-S5.ZIP › Supplementary Figures/Figure S1.pdf]

1 ~ 2 Hour

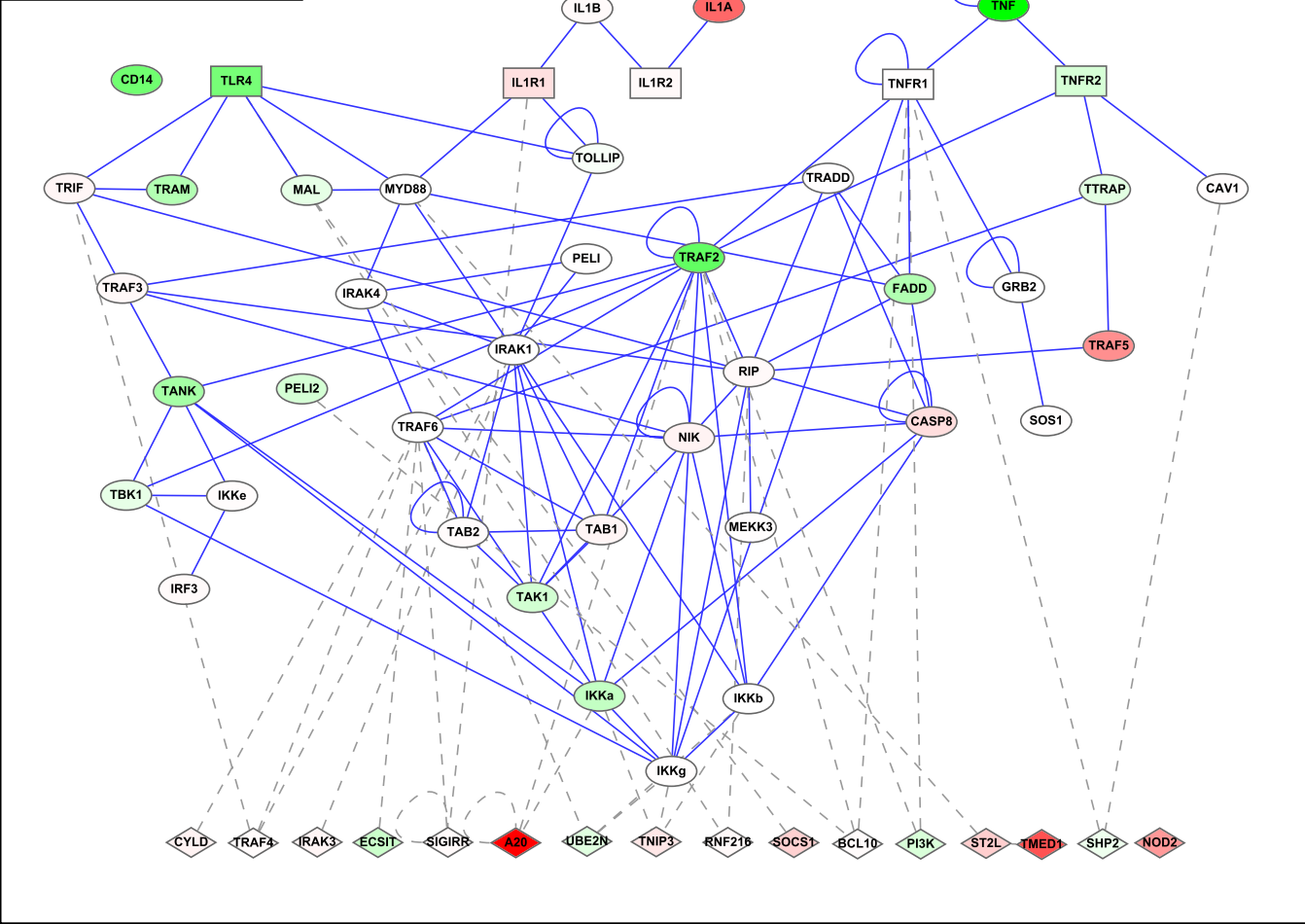

Supplement: Additional file 5 — Supplementary Figures. Complete time series diagrams of the refined PPANs under TNFα stress from 0 to 8 hour are shown in Supplementary Figures. [file 1755-8794-3-19-S5.ZIP › Supplementary Figures/Figure S2.pdf]

2 ~ 3 Hour

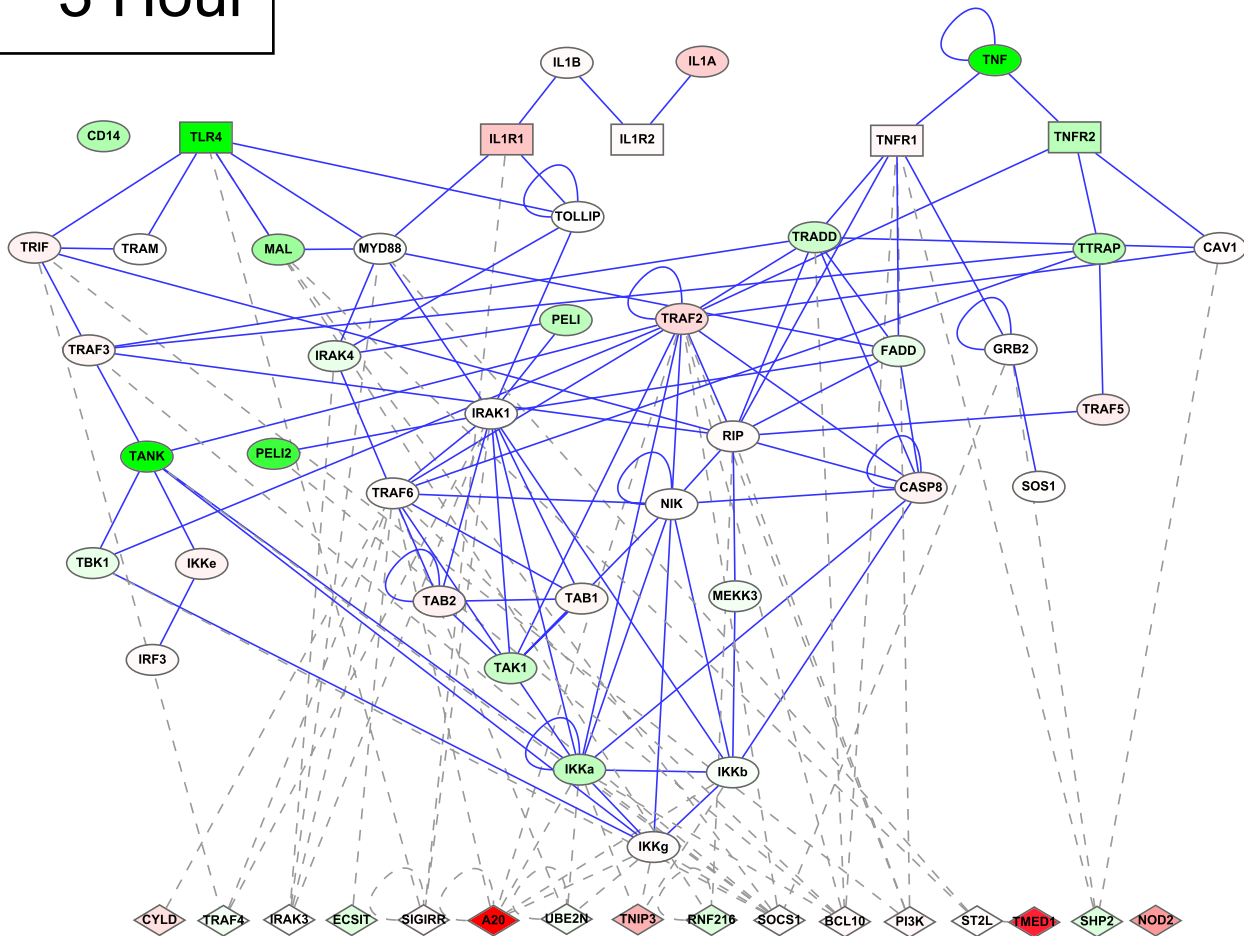

Supplement: Additional file 5 — Supplementary Figures. Complete time series diagrams of the refined PPANs under TNFα stress from 0 to 8 hour are shown in Supplementary Figures. [file 1755-8794-3-19-S5.ZIP › Supplementary Figures/Figure S3.pdf]

3 ~ 4 Hour

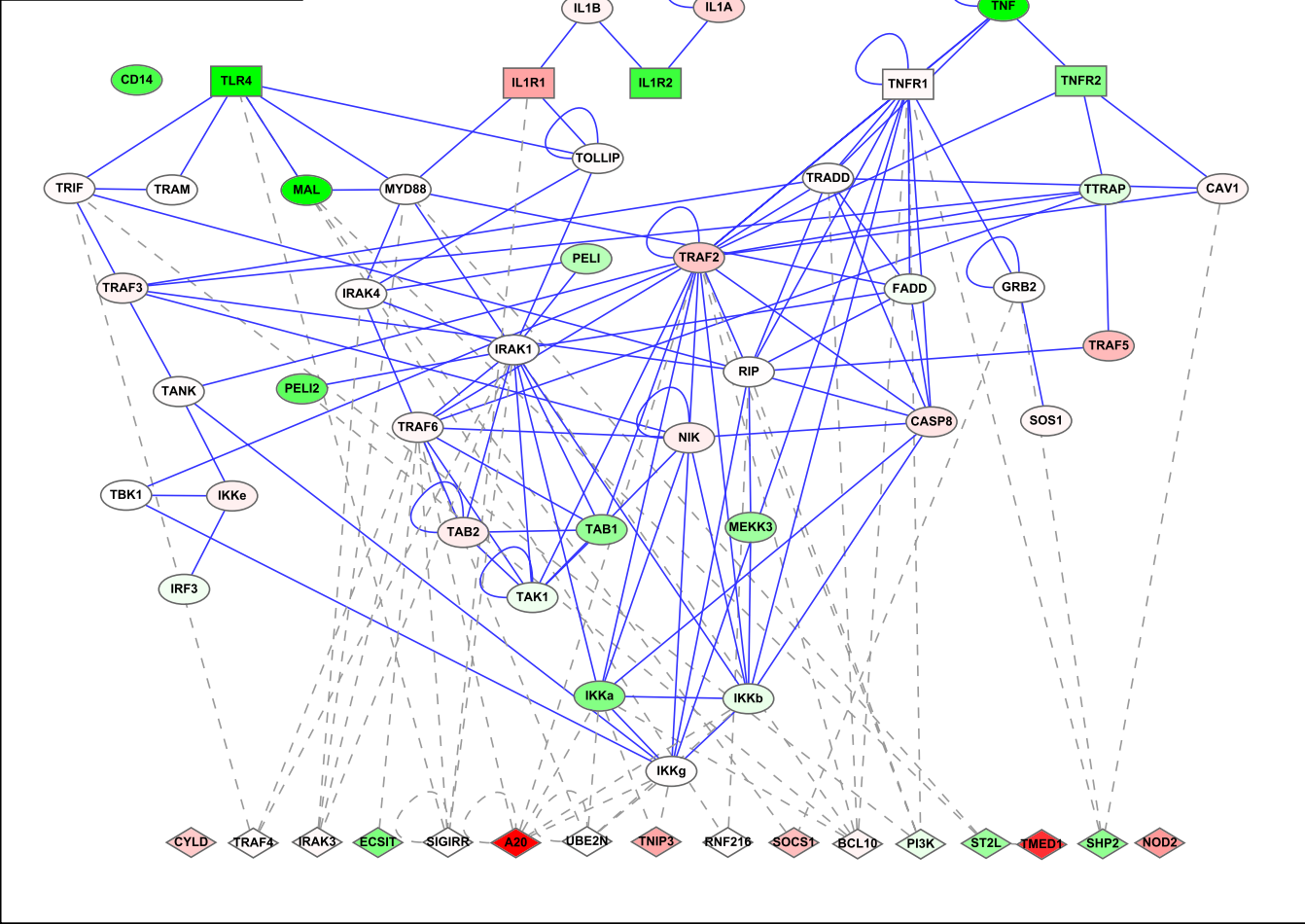

Supplement: Additional file 5 — Supplementary Figures. Complete time series diagrams of the refined PPANs under TNFα stress from 0 to 8 hour are shown in Supplementary Figures. [file 1755-8794-3-19-S5.ZIP › Supplementary Figures/Figure S4.pdf]

4 ~ 6 Hour

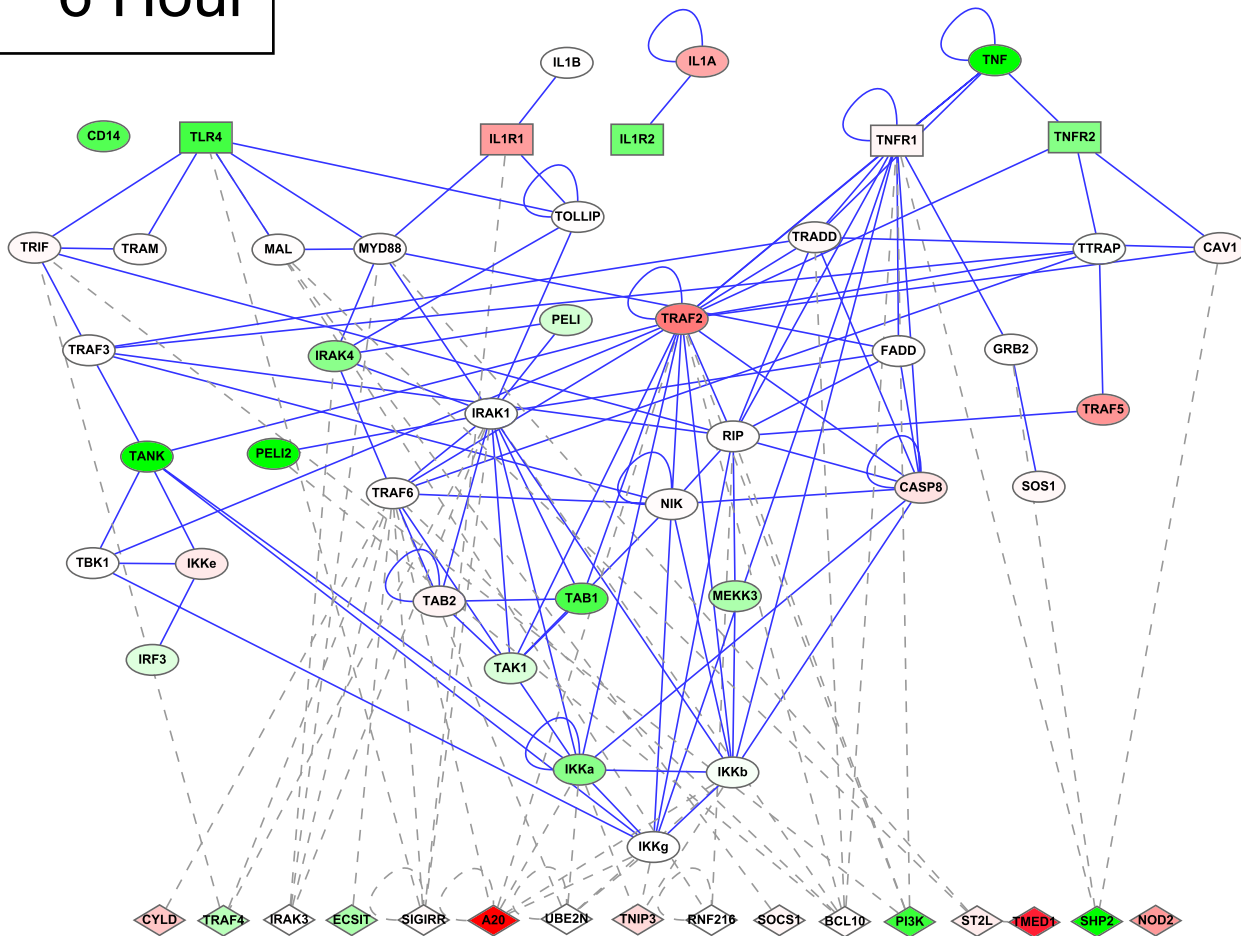

Supplement: Additional file 5 — Supplementary Figures. Complete time series diagrams of the refined PPANs under TNFα stress from 0 to 8 hour are shown in Supplementary Figures. [file 1755-8794-3-19-S5.ZIP › Supplementary Figures/Figure S5.pdf]

6 ~ 8 Hour

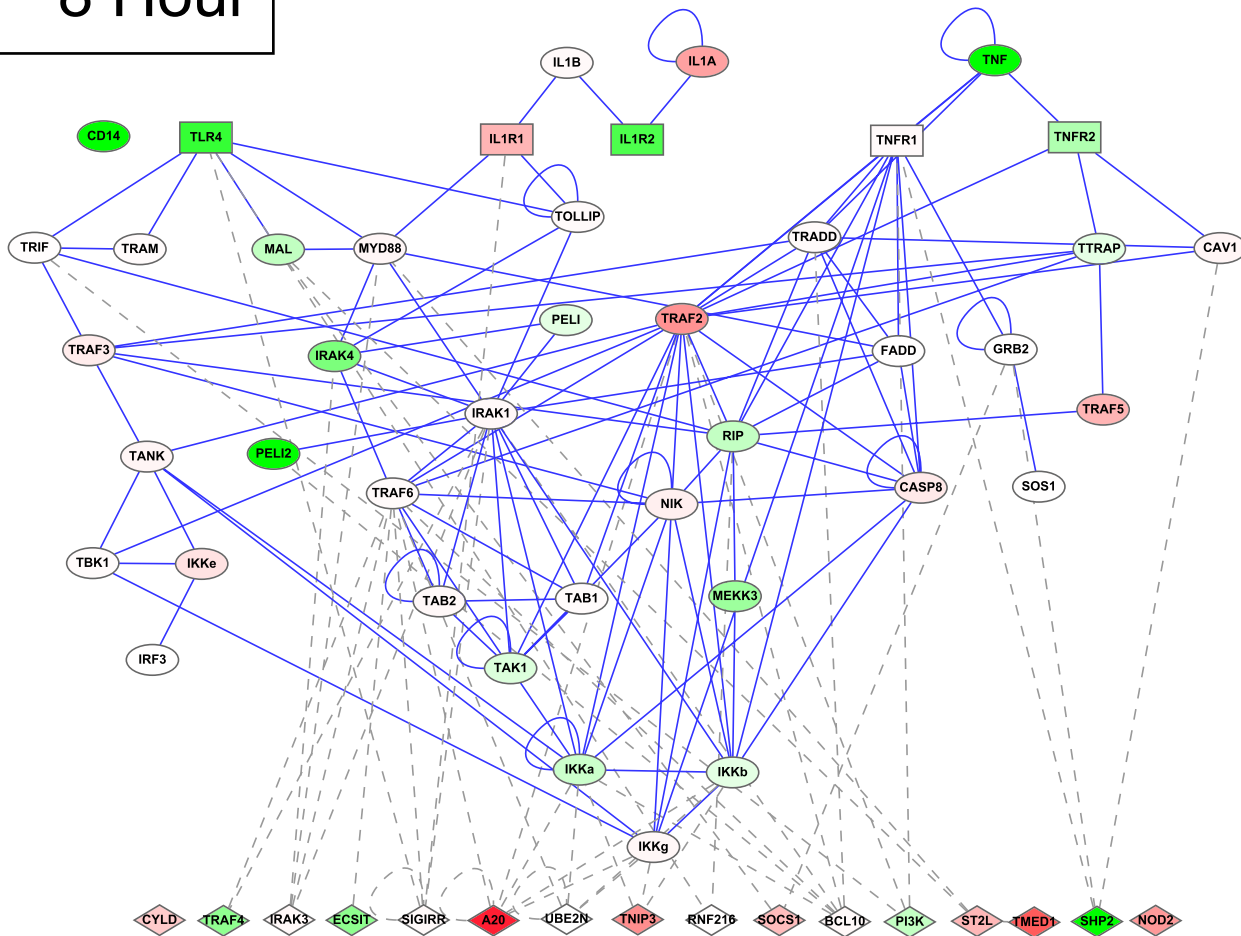

Supplement: Additional file 5 — Supplementary Figures. Complete time series diagrams of the refined PPANs under TNFα stress from 0 to 8 hour are shown in Supplementary Figures. [file 1755-8794-3-19-S5.ZIP › Supplementary Figures/Figure S6.pdf]
